# An Examination of the Condition of the Teeth of Certain Pre-Historic American Races

**Published:** 1883-10

**Authors:** W. C. Barrett

**Affiliations:** Buffalo, N. Y.


### Independent Practitioner.

Vol. IV. October, 1883. No. 10.

### Original Communications.

### AN EXAMINATION OF THE CONDITION OF THE TEETH OF CERTAIN PRE-HISTORIC AMERICAN RACES.

BY DR. W. C. BARRETT, BUFFALO, N. Y.

In the consideration of the Etiology of Dental Caries it is necessary to inquire into its history, because a disease which is entirely modern in its origin must necessarily be due to some changed condition under which the human race is now laboring, and this new situation being duly examined may afford a clue to the altered pathological state of the teeth of the present day. It has been urged with much ability and seeming consistency that dental caries is due to the changed habits of man; to our modern artificial methods of living; to an unnatural diet; to improper preparation of the food; to the removal of necessary ingredients from otherwise wholesome sustenance; to vicious nutrition through improper habits; to a perversion of neural currents through an unnatural mode of life; in short, to a departure from the state of nature in which man was made to live. If this be true, we have only to go back and examine the skeletons of those who were guilty of none of these things; who lived directly on the breast of mother nature; who ate their food with little or no preparation by fire; who devoured all there was of it without the removal of certain portions through the aid of machinery; who were not subjected to the modern abominations of corsets, swallow-tailed coats, stove-pipe hats, or other articles of modern clothing; who were free from the poisonous fumes of box stoves and modern furnaces; upon whom the winds of heaven blew untempered at all seasons of the year; who knew no doctors, drugs or dosing, except the simple remedies provided by a bountiful nature; who, to sum up all, were delivered from the destroying influences of a modern civilization: if this be true, I say, the remains of the happy people who lived in the blessed time of pre-civilization will indicate their freedom from the diseases which are so destructive to-day.

In the summer of 1882 I chanced to visit the Peabody Museum of Archæology, at Cambridge, Mass., and was so impressed by what I saw there that I made the firm resolve to return when I had more leisure, and make a more careful study of the archæological treasures therein contained, with a view of obtaining more light upon the vexed question of the etiology of dental diseases. Accordingly, in June last, I went to Cambridge and did what I could in the limited time at my disposal, and I propose in this article to give the results of my visit. I found, however, that I had devoted but a small portion of the time necessary to even a cursory examination of the collection. I propose at some time to continue the work, and hope to be able to present something yet more definite and conclusive.

Everything connected with the subject which I wished to investigate was placed at my disposal by the courteous curator, Mr. F. W. Putnam, who is so well known in the ethnological world. I found that I was treading virgin soil, for although this museum is situated at that great educational centre, Cambridge, no one had yet attempted to make any study of the teeth there.

The upper floor of the museum contains nearly two thousand skulls, and the collection is especially rich in the remains of prehistoric American races. There are many skulls of moderns, but my attention was chiefly directed to those of the North American Indian, to those of the ancient mound-builders of the Tennessee region, to skulls found in ancient Mexican caves, to some from California, to a large number obtained from ancient burial places near Ancon, Peru, to some other South American skulls, and to a collection of early Sandwich Island skulls. Besides this, I examined, as far as time permitted, some Roman skulls of the early Christian period.

It will be observed that with the exception of the Roman skulls, and perhaps a few of the north-western Indians, the people of whom these bones were relics lived before the time of contact with the whites, and were therefore pre-historic. They were not contaminated by the vices of our modern civilization, but lived a life that was as near the typical natural one as the most conservative fogy could desire. Some of them had a climate that was as near perfect as this globe affords. Nearly all of them lived in regions that furnish the most prodigal display of natural nutritious foods. The diet of some must have been almost exclusively vegetable, while the North American races existed mainly by the chase, and their food was mostly animal. Some of the races lived in huts formed by the branches or bark of trees, some in natural or artificial caves, while others had their habitation in a snowless and rainless region, and hence needed no protection from the weather, and their days and nights were passed almost exclusively in the open air. They had no mills to grind and bolt their grain into flour, and they were destitute of the condiments and palate stimulants of the present day. They drank no spirituous liquors, and only a portion of them used tobacco, and then only to smoke it in great clay pipes which must have extracted all the nicotine by absorption. They were clothed either in the skins of wild beasts, or the vegetable fabrics which their limited manufacturing skill enabled them to make, while some of the tribes were probably as ignorant of raiment as Adam and Eve before that wonderful serpent interviewed them.

Here were various races of people who were quite free from the "vices of civilization," who lived in that happy primeval state of which poets love to sing, and some of our retrospective modern dentists to preach. Surely, if happiness, innocence and freedom from disease are to be found—

"The heart that is humble might hope for it here."

What did I find? Allow me to say that I went there a believer in the modern origin of dental lesions. I was firm in the faith that tooth-ache and stomach-ache and most other aches are due to our sins; that the human race is gradually deteriorating, and that man as he departs further and further from nature's simplicity and surrounds himself more and more with the concomitants of a high civilization, as his life from generation to generation becomes increasingly artificial, so do his bodily ills increase, and diseases multiply in number and virulence. I had long been accustomed to tell my patients that were it not that the dentist's skill kept pace with the alarming increase of dental diseases, and were the law of heredity to continue in full geometrically-progressive force, the human race must in a very few generations become edentulous. These theories were the result of my own reasoning, evolved from my inner consciousness, the fruits of pure logic, the results to which indisputable mathematical equations must infallibly lead. Like many another man whose theories are the results of chamber toil, when I put myself face to face with the records of actual fact I found that my fine-spun hypotheses would not hold water, and I dropped them at once, and again resolved for the thousandth time to speculate less and to examine more.

With the exception of the syphilitic diseases, there is not known to-day an oral trouble which all these nations were not familiar with, in its most exaggerated form. I say with the exception of syphilis, for in all those skulls I found but one that bore unmistakable traces of that dread scourge, and it was not at all certain that this was not intrusive and belonging to a later period. This anomaly was found in a cave in Mexico, but it so differed in type from those found with it, and the cave bore such evident marks of having been opened in historic times, that I strongly suspected this of being modern.

In the jaws of all the people whose skulls I examined, I found traces of all the diseases known to modern dentistry. There was caries of the most formidable character, black, white and brown. There were the marks of abscesses which devastated great regions of tissue. There was necrosis which had caused the loss of great sloughs of bone, though I must admit that necrosis was more rare than most other oral diseases. There were great masses of tartar enveloping all the teeth in the jaw. There were indications of all kinds of inflammation of soft tissues. There were exostoses and hypertrophies, absorptions and malformations, denudations and abrasions, exfoliations and irregularities. I thought to myself as I held in my hands the evidences of all this disease and suffering, what could these poor creatures do, tormented as they were without hope?—for there were no dentists in those days. Decayed teeth must remain until they could be removed with the fingers, for the primitive people knew not the blessedness, the comforting tenderness of modern forceps. I tried to picture to myself the victim of this terrible decay, that had in some instances destroyed every tooth in the head. I tried to imagine him brooding in silence and gloom over his misery; suffering in patience the exquisite torture with which to his untutored mind the Great Spirit had in infinite mercy and loving-kindness, or in dark wrath and sullen judgment, visited his weak creations, or in unexpected paroxysms of anguish raising a whoop that shook the forest and caused his dependent family to quake with fear, and I said, "Poor fellow! your lot *was* a hard one. Why the mischief didn't you have it filled—with amalgam if you could not afford gold?" And then I recollected that he was deprived of that inestimable last gift of a good Providence, the modern dentist, and could only suffer and groan and howl and kick the furniture about, and try old-women quack-nostrums until he felt better.

There were fewer irregularities among the teeth of these people than are known at the present day. Their jaws, especially those of the mound-builders, were broad and well developed, giving indications of a muscular race. Nevertheless, bad cases of irregularity were not unknown. The teeth of the Southwestern Indians were the most worn by use, and it was not unusual to find whole dentitions worn completely down to the gums. The Sandwich Islanders had the handsomest dentitions that I ever saw. Their teeth were almost uniformly as white as ivory, and as regular and even as the teeth of a horse. But they were, as a people, sadly afflicted with caries.

There were a number of points which I desired especially to note, and the results of these were usually disappointing to me.

The number of wormian bones did not greatly exceed those found in skulls of to-day, though some of them were unusually large. In one skull, from the mound-builders, the parietal bones were divided by a suture extending clear across them, just above the summit of the squamous portion.

There were but three cases of persistent frontal sutures in adults, all from the Tennessee mound-builders, the others differing not at all in their apparent development from the skulls of moderns.

The number of the teeth has not changed. There was about the usual number of instances of apparently rudimentary wisdom teeth, and supernumerary teeth were not lacking. There were cases of entire lack of certain teeth, the non-appearance of wisdom teeth in adult and even senile jaws being most common. I also found a number of cases of non-eruption of cuspid teeth. In some cases the wisdom teeth had been erupted while the canines were absent. I found such abnormalities as the appearance of a cuspid between the two bicuspids, and malposition of incisors. Usually, however, all the bicuspids and molars occluded perfectly. I found impacted teeth and teeth lying transversely or longitudinally in the jaw. I found some cases of narrow jaws, where there was not room for the eruption of wisdom teeth, which must have caused great annoyance and pain to the possessor.

Perhaps a typical semi-tropical race was that which inhabited ancient Peru. At just what period this people lived, we have no knowledge, but it was some time before the advent of the whites upon this continent. They were a well-built race of people, physically, and had made some advances in manufactures, being in this respect the superiors of the North-American races. That they were a patient, rather industrious, laborious people, the works which they have left behind them show. They had well developed jaws, the lower one especially being broad and strong. Their teeth were large and fine, generally of a yellowish color, but they were badly troubled by dental diseases. Manifestly they took no care of them, for they were frequently encrusted with tartar. The dentitions of the adults almost uniformly gave indications of wear, many of them being extensively abraded.

I had not opportunity or time to make careful statistics, but the following figures will give an approximate idea of the condition of the teeth of the early inhabitants of Ancon, Peru.

Of the skulls of adults of various ages, I made examinations, more or less careful, of two hundred, taking them as they came, and making no selection. I think they were a fair average of the whole. Of these, seventy-four had lost some of their teeth during life. Forty-three only, so far as I could discover, possessed a perfect dentition and gave evidence of healthy mouths. One hundred and two presented indications of oral diseases. The remainder were in such a state that it was impossible to determine their condition at death. Thirty-one showed extensive absorption of the alveolar process, due probably to various inflammatory conditions. In thirteen cases I found the marks of deep alveolar abscesses which had caused extensive wasting of the osseous tissue. How many others there were, not of sufficient severity to leave indelible traces upon the bone, it is impossible to say. In two mouths I found supernumerary teeth, and in two there were exostoses so great as to be visible. It will readily be understood that, as the remaining teeth were usually firm in their sockets,ordinary hypertrophies were undiscoverable without mutilation of the specimens. In fifteen cases there was no sign of the presence of wisdom teeth, nor of their loss. Seventy-two dentitions were extensively worn, some of them nearly even with what must have been the border of the gums In five instances the wisdom teeth were in appearance but rudimentary. In eighteen cases the wisdom teeth occupied an abnormal position through lack of room for their development, and this, notwithstanding the usually massive character of the maxillæ. There were four cases of decided irregularity, not counting unimportant deviations from the normal. There were scarce any dentitions that were entirely free from tartar, and some of the mouths contained large masses of calcareous incrustations. There was at least one case of an almost complete calcification of the pulp. How many others, only an examination of the pulp chamber could determine.

Of the ancient mound-builders of Tennessee I examined the skulls of seventy-five. Of these, twenty-seven could be set down as probably free from dental diseases. Thirty-two had carious teeth. There were seven cases of extensive alveolar abscess. Twenty-two had lost teeth during life, and the consequent absorption had obliterated all trace of their existence. Twelve cases showed extensive absorption about existing teeth. There were two cases of visible exostoses, two apparently rudimentary wisdom teeth, and three irregular dentitions. Only seventeen showed depositions of tartar, and but thirteen gave evidences of much wear.

It will be seen that the teeth of the mound-builders were much more free from calcareous incrustations, and less worn than those of the inhabitants of Peru. This was doubtless owing to their living more largely upon an animal diet. There were less cases of irregularity because they were a larger and more muscular race, with broader, stronger jaws, but the proportion of diseased dentitions did not vary greatly.

The teeth of the Sandwich Islanders were much more beautiful, and instances of irregularity were very rare. But the same prevalence of caries and other dental diseases was observable, though in probably not as great proportion. I was unable in the limited time at my disposal to attempt to gather any statistics of the skulls of this people, or of those of the few Mexicans, California Indians, and Patagonians in the collection. It must suffice here to say that all showed the presence of the same diseases which we are called upon to treat to-day, and the proportion, while undoubtedly varying among the different nationalities, was sufficiently large in all cases to have given ample employment to a fair sprinkling of dentists, had they existed.

What is the conclusion of this whole matter? It is that dental caries and oral diseases are not the results of modern civilization. That they are not wholly due to errors of diet, nor to the use of tobacco, or condiments, or to any peculiar manner of preparing food, nor do they have their origin in perverted neural currents and the general deterioration of mankind, since they have accompanied man, so far as we can trace, through all the gradations of his development.

The further consideration of the subject I leave for the future, only insisting that while I do not in this paper of a necessarily hurried preparation make pretension to minute exactness, the general truth of the observations made and the approximate accuracy of the statistics given, I will stoutly maintain. Any one who considers the conditions under which such researches are made, and the care which must be exercised to prevent accidental mutilation of the treasured specimens, the consequent impossibility of making intimate inquiry into the internal condition of the teeth and jaws, and the necessary absence of microscopical investigation of the tissues, will understand that little more than general results can be expected from such an examination as that which I have here reported.

